# Screening of dysphagia in geriatrics

**DOI:** 10.1186/s12877-022-03685-1

**Published:** 2022-12-19

**Authors:** Ayatallah Raouf Sheikhany, Sahar Saad Shohdi, Azza Adel Aziz, Omnia Abass Abdelkader, Aisha Fawzy ِAbdel Hady

**Affiliations:** Phoniatric Unit, Otolaryngology Department, Faculty of Medicine, Cairo University, Giza, Egypt

**Keywords:** Oropharyngeal dysphagia, Geriatric population, Non-complaining aging subjects

## Abstract

**Background:**

The oropharyngeal dysphagia is an underestimated symptom with various causes in the geriatric population. Clinical presentation is often insidious and dysphagia symptoms are seldomly mentioned by elderly patients although causing many life-threatening complications. The aim of this work was to introduce an easy applicable tool to be used by the caregivers and general practitioners for screening of dysphagia in geriatrics for early detection of at risk individuals.

**Methods:**

A sample of 200 Egyptian Arabic-speaking elderly patients (65 years or older) not complaining of dysphagia was recruited from nursing homes in Greater Cairo Area. They or their caregivers completed the designed screening tool, including; the designed questionnaires of dysphagia manifestations and eating habits. General, oral motor and bedside evaluation were also performed. In addition to filling in the EAT10 questionnaire and FEES that was performed for only suspected cases for the purpose of validation of the screening tool.

**Results:**

The dysphagia manifestations questionnaire was significantly correlated with EAT 10 with p value of 0.001. It was correlated in some of its aspects with FEES showing quite reliability with p values’ range between 0.012 and 0.044. The Questionnaire of eating habits reliability of r- value of 0.568 slightly exceeding EAT10 reliability of r -value of 0.721 in the subjects under study. The cutoff point of total score of the dysphagia manifestations was > 5, with a sensitivity of 17.65% & a specificity of 94.20%. The cutoff point of total score of the bedside evaluation was ≤ 1 with a sensitivity of 66.9% & a specificity of 56.9%.

**Conclusion:**

the use of this easy applicable screening tool managed to suspect and later on diagnose cases with oropharyngeal dysphagia in non-complaining aging subjects.

## Background

Geriatrics is the medical care of the elderly, an age group that is difficult to precisely define, however age of 65 years or more is the age that is often used [1, 2]. It is predicted that by the year 2050, 25% of the population in the developing countries will be accounted for by people aged 65 years or older, As the increase in longevity over the past 50 years is being reported, understanding changes in the physiology with aging and the unique challenges this population faces is needed [3].

Dysphagia occurs mainly in elderly people, usually beginning at the age of 45 years. This is called presbyphagia, which results from multiple age related changes in the anatomy of head and neck as well as changes in the neural and physiologic mechanisms controlling swallowing. In addition, the prevalence of diseases increase with aging, and dysphagia is known to be a common confiding of many diseases or their treatments [4]. Dysphagia may be primarily related to the aging physiology, or secondary to neurologic and neuromuscular disorders which are found to be correlated with dysphagia [5]. Post stroke dysphagia leads to complications such as pneumonia, malnutrition, dehydration, and increased duration of hospitalization [6]. Dementia is linked with dysphagia. Forty-five percent of patients with dementia experience difficulty with swallowing; therefore, they are exposed to malnutrition [7].

Oropharyngeal dysphagia in old age has been recognized as a geriatric syndrome [8]. Dysphagia is related to sarcopenia and this highlights the fact that loss of muscle mass and power is under the umbrella of the universal phenomenon of aging [9]. As specific exercises are available to strengthen muscles in oral motor difficulties, it is particularly important that professionals caring recognize early dysphagia signs [10]. Oropharyngeal dysphagia is a main cause of anorexia in geriatrics, and weight loss can aggravate dysphagia [11]. Dental diseases are common leading to change of the composition of oropharyngeal flora causing pneumonia in elderly patients in nursing home [12].

Complications of dysphagia such as Malnutrition, dehydration, in addition to aspiration pneumonia affect 7% to 13% of people aged 65 years or older [13]. Individuals having cognitive dementia or Parkinsonism or those who are in assisted-living facilities are specifically vulnerable to dysphagia; Up to 50% of the latter group suffers from swallowing difficulties [14].

Dysphagia significantly impacts quality of life, with social and psychological consequences. Dysphagia in the elderly can be misidentified as a normal aging by not only physicians but also the patients themselves, thus remains undetected [3]. Additionally, the workup of dysphagia is difficult, as it requires a multidisciplinary approach with involvement of multiple specialties as primary care physicians, geriatricians, otolaryngologists, neurologists, gastroenterologists, phoniatricians and speech language pathologists, occupational therapists and nutritionists. With dire consequences and mortality, all old patients should be evaluated for swallowing difficulty [15].

With elderly people electing to stay in their homes for as long as possible, predisposing factors such as advancing age may compromise procurement, preparation of food and eating and have a detrimental impact on nutrition status. Elderly may be more nutritionally vulnerable and lack the physical reserve; so it is recommended to screen swallowing problems in older persons [16].

The question of ‘What about swallowing?’ is repeatedly used without performing any extra standardized testing. If the patient did not give a positive response, so no further recommendation for swallowing testing to be carried out. Coughing, choking, food sticking in the throat sensation after deglutition and respiratory problems are aspects of oropharyngeal dysphagia. The usage of a single question on swallowing instead of a detailed questionnaire like the EAT-10 might cause under-diagnosis of at risk patients to develop dysphagia [17].

To date, there is limited literature supporting a strong association between aging and swallow outcomes. However, among older adults in long term care it was found that there is an association between observable signs of swallowing difficulty and reduced food consumption. Therefore, exploring associations among aging, dysphagia and malnutrition appears warranted. In Egypt, the screening for risk of oropharyngeal dysphagia is not mandatory and knowledge of prevalence of malnutrition in elderly adults is lacking. So this study aimed at investigating the magnitude of dysphagia and subsequent complications in older adults.

The aim of this work was to introduce an easy applicable tool to be used by caregivers and general practitioners for early detection of dysphagia in non-complaining geriatrics to pick up the risky individuals and refer them as early as possible to receive the needed intervention.

## Methods

### Study population

Subjects under study were recruited from five nursing homes in Greater Cairo Area, each had an estimate of 60 elderly people. A number of 300 elderly people were interviewed then subjects under study were selected based on the inclusion and exclusion criteria. The study was conducted from December 2018 to March 2020. The study was in accordance.

with the Declaration of Helsinki. All experiments were carried out in accordance with relevant guidelines and regulations.

Inclusion criteria include elderly people aged 65 or more of both genders not complaining of dysphagia living in Greater Cairo Area. The participants were selected to be literate (can read and write) or at least has a literate caregiver.

Exclusion criteria include participants presented with dysphagia secondary to neurological insults or had a previous history of dysphagia and participants with severe cognitive impairment.

## Methodology

In order to obtain a comprehensive screening of dysphagia in the aging population. The participants underwent the three steps of the designed screening protocol of this study including:

1. Two specifically designed questionnaires for dysphagia

A- Questionnaire of dysphagia manifestations.

B- Questionnaire of eating habits.

2. Screening of general and oral motor skills
3. A complete bed side evaluation of dysphagia.
4. After approval of the nursing homes, elderly people under study were informed about the purpose of the study, a written consent from them or their caregivers was obtained. The subjects under study were exposed to the following:
5. Subjects were interviewed by two phoniatricians; a trained junior and a senior staff of Phoniatrics, and they or their caregivers were asked to fill in two questionnaires of "dysphagia manifestations and eating habits". The questionnaires were specifically designed in the study by a number of 5 qualified phoniatricians who had sufficient experience and dealt with many dysphagic cases of different etiologies. The items were collected in light of questionnaires previously done by Baijens et al. [10], Belafsky et al. [18], and Wallace et al. [19], in addition to the common symptoms presented by dysphagic patients specifically in the geriatric population found in the literature and through clinical experience of phoniatricians performing the study.

The questionnaire of *dysphagia manifestations* is formed of 14 questions to get an overall view about dysphagic manifestations such as: anorexia, weight loss, food accumulation in the mouth, drooling, nasal regurgitation of fluids and food, cough on fluids and food, the need to drink water to get easy swallow, eating in pieces & frequent swallows per bolus. If there was a complaint, the subject would get a score of 1 but if there was no problem or complaint, the subject would get a score of 0.

The questionnaire of *eating habits* include number of meals / day, duration of meal, consistency of meals, the condition of surrounding environment as regards being distracting or prepared, the need to use any specific food utensils, the need of any assistance in eating and the easiest and the most difficult type of food.

Then all the participants in the current study were subjected to screening of the general *condition and oral motor skills* done by the caregivers using a checklist that included the following items: presence of attention difficulty, cooperation problem, breathing incoordination, language problem, voice change, problem with understanding orders, imitation disability, posture problem, and difficulty in speech clarity, presence of salivation, palatal elevation problem, problems with lips symmetry, closure and deviation, teeth if they present, absent or had artificial teeth, problems with jaw size, symmetry, deviation and movement, gingival problem, absent gag reflex and problems with tongue size, muscle power and movement as guided by Skue et al. [20] and Takeshi et al. [21].

If there was a defect or a problem, the subject would get a score of 1 but if there was no problem, the subject would get a score of 0.

All the subjects then underwent a *Bedside evaluation* which is helpful in detecting the suspected cases that need instrumental evaluation of dysphagia. The subjects were given by their caregivers 150 ml of fluids of different consistencies (water as an example of thin fluids and pudding as an example of thick fluids). They were instructed to drink without interruption. Each subject was observed for 1 min to detect if there was any kind of salivation, accumulation of fluids in mouth, cough or any voice changes.

Then all subjects underwent a trial of feeding using different consistencies. Solids ( biscuits, cake or bread), semisolids ( yogurt) and mixed consistency (a slice of orange). Each subject was observed for 1 min to detect if there was any kind of salivation, difficulty in chewing, accumulation of food in mouth, cough or any voice change. If there was any observed abnormality, the subject would get a score of 1 but if there was no problem, the subject would get a score of 0 as guided by Sheikhany et al. [22].

A pilot study was applied on 20 old-aged persons to test the applicability of the screening tool, to find if there were any items that weren’t easy comprehended by the subjects or by their caregivers and to determine the duration of the screening. The screening tool was found to be easily comprehended and need very minimal instructions by the assessor and the duration taken was from 25 to 30 min.

### Validation of the screening tool

For the purpose of validation of the screening tool, EAT 10 [23], and FEES (Fiberoptic Endoscopic Evaluation of Swallowing) were used.

Only suspected subjects under study who gave a history of choking or weight loss by the dysphagia manifestations and eating habits questionnaires with findings of effortful swallowing or chocking detected by screening using bedside evaluation underwent the step of assessment using FEES.

*EAT-10* was translated to the Arabic language by Farahat and Mesallam [23]. The Arabic version of the EAT-10 was applied to all subjects under study to correlate its results with those obtained from the questionnaires designed in the study. EAT-10 is a self-reported validated questionnaire that assesses perception of swallowing difficulty [24]. It can be filled in by the participants themselves or their caregivers. It is used to evaluate dysphagia risk. Increase in the EAT-10 score is indicative of increased dysphagia risk or swallowing difficulties [25]. Previous studies such as Belafsky et al. [18], stated that EAT-10 score ≥ 3 is suggestive of dysphagia and need of further assessment to detect the cause of swallowing difficulty.

Twenty subjects underwent the FEES as their scores on the questionnaires and beside evaluation were suspicious and indicative of dysphagia. FEES is considered the method of choice for studying swallowing disorders as it is easy to use, very well tolerated; allow bedside examination and economic to detect the underlying oropharyngeal breakdown in the swallowing mechanism. It has few reported complications as discomfort; gagging and/or vomiting that can be observed during practice as well as seldomly more severe complications such as laryngospasm or vasovagal syncope [26]. FEES was conducted to objectively evaluate if there is a breakdown in the oro-pharyngeal stage of swallowing. The subject was seated in an upright position on a chair in the swallowing clinic. All steps of the procedure were explained to the subject and his caregiver. An examination tray was then placed next to the caregiver on the left side of the patient while the assessor was seated on the right side.

The endoscope was then placed inside the subject’s nose and passed to the oropharynx to allow static ‘ structure’ and dynamic ‘ physiology’ evaluation of the oro-hypo-pharyngeal and laryngeal structures and the pre-swallowing and post-swallowing findings. The same consistencies used in the bedside evaluation were given by the caregiver and the swallowing process for each was observed. Findings reported if there was: velopharyngeal valve incompetence, pharyngeal mobility problem, premature spillage, residue, penetration, aspiration and glottis closure problem.

Each subject was scored 1 for the presence of any abnormality, and 0 for absence of the finding. After collecting the data, they were tabulated and statistically analyzed.

### Statistical analysis

Statistical Package for Social Science (IBM SPSS) version 20 was used for statistical analysis [27]. Qualitative data were presented as numbers & percentages. The mean, standard deviations and ranges were used for the quantitative data. Parametric distribution and median with inter quartile range (IQR) were applied for the quantitative data with non-parametric distribution**.**

Chi-square test was used in the comparison between two groups with qualitative data. Fisher exact test was applied when the expected count in a cell was found to be less than 5 [28]. In the same group, Spearman correlation coefficients were applied to assess the relation between two quantitative parameters. The confidence interval (CI) was set to 95%. The margin of error accepted was 5%. The p-values’ significance was considered as the following: *P* > 0.05: Non significant (NS), *P* < 0.05: Significant (S) and *P* < 0.01: Highly significant (HS).

For Cronbach’s α reliability, when α is 0.0to 0.20, the reliability is described as less reliable, > 0.20 to 0.40 is considered rather reliable, > 0.40 to 0.60 is considered quite reliable, > 0.60 to 0.80 is considered reliable and > 0.80 to 1.00 is considered very reliable.

## Results

Three hundred Egyptian elderly people were interviewed from 5 nursing homes in Greater Cairo area and only two hundred elderly people were included in the study, with an age range of 65 to 78 years old and a mean age of 71.5. Males represented 63.4% of the sample under study with a mean age of 73.32 while females represented 36.6% of them with 70.41. They were all of moderate socioeconomic standard. Almost 70% of them did not have a spouse (widowed, divorced, or never married). They were literate except for 20% of them whom the assessors and/or their caregivers helped them fill in the questionnaires. None of them had a current or previous history of swallowing difficulty or any medical condition that might cause dysphagia.

The most encountered dysphagia symptoms were in 36.5% of the elderly people under study ‘coughing on fluids’, in 26.5% ‘sticking of food in the throat’, in 25.5% ‘the need to drink water to swallow’, while in 22.5% ‘ the need to cut food into small pieces’ and in 21.5% ‘ need for multiple swallows’. The least percentages were for the presence of salivation in 12.5% of the subjects and weight loss in 15% of them. The subjects under the study didn’t experience any difficulty to initiate swallow or any nasal regurgitation of food as shown in Table 1.

**Table 1:** Results of dysphagia manifestations questionnaire

		**No (total No:200)**	**%**
Dysphagia		37	18.5%
Anorexia		36	18.0%
Weight loss		30	15.0%
Salivation		25	12.5%
Accumulation of food in mouth		31	15.5%
Sticking of food in throat		53	26.5%
Difficulty to initiate swallow		0	0.0%
Nasal regurgitation of food		0	0.0%
Nasal regurgitation of fluids		6	3.0%
Cough on food		37	18.5%
Cough on fluids		73	36.5%
Need water to swallow		51	25.5%
Multiple swallows		43	21.5%
Need to cut food into small pieces		44	22.0%
Total score	Mean	2.34	
	Range	0 – 11	
	Median	1	

Table 1 showed that the most encountered dysphagia symptoms were in 36.5% of the elders under study ‘coughing on fluids’, in 26.5% ‘sticking of food in the throat’, in 25.5% ‘the need to drink water to swallow’, while in 22.5% ‘ the need to cut food into small pieces’ and in 21.5% ‘ need for multiple swallows’. The least percentages were for the presence of salivation in 12.5% of the subjects and weight loss in 15% of them. The subjects under the study didn’t experience any difficulty to initiate swallow or any nasal regurgitation of food

67% of subjects under study had 3 meals a day while only 33% of them had 2 meals a day and the meal duration for all of them was less than 30 min. 66% of the subjects could eat all consistencies of food and 50% of the subjects needed prepared environment during mealtimes. None of them were surrounded by expert caregivers nor used special tools for eating and none needed help during mealtime. 61% of the subjects had difficulty with at least one food consistency, 97.5% of the subjects under study had difficulty in eating solids while semisolids were the easiest consistency for them as shown in Table 2.

**Table 2:** Results of eating habits questionnaire

		**No (total No:200)**	**%**
No. of meals	Two	66	33.0%
	Three	134	67.0%
Duration of meal	< 1/2 h	200	100.0%
	> 1/2 h to 1 h	0	0.0%
	> 1 h	0	0.0%
Consistency of meal	Fluids	0	0.0%
	Semi solids	68	34.0%
	Solids	0	0.0%
	All	132	66.0%
Eating environment	Prepared	100	50.0%
	Distracting	100	50.0%
Experience of caregivers	0	200	100.0%
Using special tools for eating	No	200	100.0%
	Yes	0	0.0%
Need help food time	No	200	100.0%
	Yes	0	0.0%
Presence of difficult consistency	No	122	61.0%
	Yes	78	39.0%
The easiest consistency	Fluids	2	2.5%
	Semi solids	78	97.5%
The most difficult consistency	Fluids	2	2.5%
	Solids	78	97.5%

Table 2 showed that 67% of subjects under study had 3 meals a day while only 33% of them had 2 meals a day and the meal duration for all of them was less than 30 min. 66% of the subjects could eat all consistencies of food and 50% of the subjects needed prepared environment during mealtimes. None of them were surrounded by expert caregivers nor used special tools for eating and none needed help during mealtime. 61% of the subjects had difficulty with at least one food consistency, 97.5% of the subjects under study had difficulty in eating solids while semisolids were the easiest consistency for them

There was breathing incoordination, language problems, voice changes, postural problems, reduced speech clarity and gingival affection in 1% of the subjects under study. 12% of the subjects under study showed small atrophic tongue and poor lips, tongue and jaw muscle power while 3% of subjects under study had tongue tremors. 47% of subjects showed missing teeth as shown in Table 3.

**Table 3:** Results of general and oral motor examination

		**No (total No:200)**	**%**
Attention difficulty		0	0.0%
Cooperation problem		0	0.0%
Breathing incoordination		2	1.0%
Language problem		2	1.0%
Voice change		9	4.5%
Understanding orders problem		0	0.0%
Imitation disability		0	0.0%
Posture problem		4	2.0%
Difficulty in speech clarity		2	1.0%
Salivation		7	3.5%
Palatal elevation problem		0	0.0%
Lips symmetry affection		0	0.0%
Lip closure affection		0	0.0%
Lip deviation affection		0	0.0%
Denture	Present teeth	75	37.5%
	Missing teeth	94	47.0%
	Appliance	31	15.5%
Jaw deviation		0	0.0%
Jaw symmetry affection		0	0.0%
Poor jaw mobility		0	0.0%
Gingival problem		2	1.0%
Absent gag reflex		0	0.0%
Small tongue size		24	12.0%
Poor muscle power		24	12.0%
Tongue tremors		6	3.0%

Table 3 shows that there was breathing incoordination, language problems, voice changes, postural problems, reduced speech clarity and gingival affection in 1% of the subjects under study. 12% of the subjects under study showed small atrophic tongue and poor lips, tongue and jaw muscle power while 3% of subjects under study had tongue tremors. 47% of subjects showed missing teeth

3.5% of subjects experienced salivation and accumulation of semisolids, solids and mixed consistency food while 6.5% of subjects showed voice change on thin and thick fluids, 27% showed difficulty on chewing solids while 52.5% had difficulty on mixed consistency as evident by frequent coughing, need to clear the throat and in some cases prolonged oral preparation phase as shown in Table 4.

**Table 4:** Results of bedside evaluation

	**No (total NO:200)**	**%**
Neck position problem on eating	0	0.0%
Mouth position problem on eating	0	0.0%
Salivation on thin fluids	0	0.0%
Salivation on thick fluids	0	0.0%
Salivation on semisolids	7	3.5%
Salivation on solids	7	3.5%
Salivation on mixed	7	3.5%
Accumulation on thin fluids	0	0.0%
Accumulation on thick fluids	0	0.0%
Accumulation on semisolids	7	3.5%
Accumulation on solids	7	3.5%
Accumulation on mixed	7	3.5%
Chewing difficulty on thin fluids	0	0.0%
Chewing difficulty on thick fluids	0	0.0%
Chewing difficulty on semisolids	0	0.0%
Chewing difficulty on solids	54	27.0%
Chewing difficulty on mixed	105	52.5%
Cough on thin fluids	21	10.5%
Cough on thick fluids	21	10.5%
Cough on semisolids	0	0.0%
Cough on solids	0	0.0%
Cough on mixed	0	0.0%
Voice change on thin fluids	13	6.5%
Voice change on thick fluids	13	6.5%
Voice change on semisolids	0	0.0%
Voice change on solids	0	0.0%
Voice change on mixed	0	0.0%

Table 4 shows that 3.5% of subjects experienced salivation and accumulation of semisolids, solids and mixed consistency food while 6.5% of subjects showed voice change on thin and thick fluids, 27% showed difficulty on chewing solids while 52.5% had difficulty on mixed consistency

All subjects under study experienced no pain on swallowing, 7.5% showed stress on swallowing, 2.5% had weight loss due to their swallowing problem, 3.5% showed cough on swallowing, 6.5% had affected eating pleasure due to their swallowing problem, 17.5% of subjects under study needed extra effort on swallowing pills and their swallowing problem interfered with their ability to go out as shown in Table 5.

**Table 5:** Results of EAT10 questionnaire

		**No (total No:200)**	**%**
Item 1 My swallowing problem cause weight loss	0	175	87.5%
	1	2	1.0%
	2	12	6.0%
	3	11	5.5%
Item 2 My swallowing problem interferes with my ability to go out for meals	0	165	82.5%
	1	2	1.0%
	2	23	11.5%
	4	10	5.0%
Item 3 Swallowing liquids takes extra effort	0	167	83.5%
	1	15	7.5%
	2	11	5.5%
	3	7	3.5%
Item 4 Swallowing solids takes extra effort	0	133	66.5%
	2	28	14.0%
	3	31	15.5%
	4	8	4.0%
item 5 Swallowing pills takes extra effort	0	165	82.5%
	2	14	7.0%
	3	21	10.5%
Item 6 Swallowing is painful	0	200	100.0%
Item 7 The pleasure of eating is affected by my swallowing	0	167	83.5%
	2	24	12.0%
	3	9	4.5%
Item 8 When I swallow, food sticks in my throat	0	153	76.5%
	2	13	6.5%
	3	34	17.0%
Item 9 I cough when I eat	0	173	86.5%
	2	21	10.5%
	3	6	3.0%
Item 10 Swallowing is stressful	0	185	92.5%
	2	13	6.5%
	3	2	1.0%
Total score of EAT10	Mean	3.74	
	Range	0–25	
	Media	0	

Table 5 shows that all subjects under study experienced no pain on swallowing, 7.5% showed stress on swallowing, 2.5% had weight loss due to their swallowing problem, 3.5% showed cough on swallowing, 6.5% had affected eating pleasure due to their swallowing problem, 17.5% of subjects under study needed extra effort on swallowing pills and their swallowing problem interfered with their ability to go out

The highest percentage (7%) was for the presence of residue, while the least percentage (1.5%) was for the presence of aspiration. 2% of subjects had incomplete glottic closure and VPV incompetence, 4% of subjects had premature spillage and poor pharyngeal mobility while 4.5% of subjects had penetration and delayed triggering as shown in Table 6.

**Table 6:** Results of FEES in the suspected group

		**No (total NO:20)**	**%**
Presence of penetration		9	4.5%
Presence of aspiration		3	1.5%
Presence of Residue		14	7.0%
Incomplete glottis closure		4	2.0%
VPV incompetence		4	2.0%
Presence of premature spillage		8	4.0%
Delayed triggering		9	4.5%
Poor pharyngeal mobility		8	4.0%
Associated		4	2.0%
Total score	Mean	4	
	Range	1–7	
	Median	4	

Table 6 shows that the highest percentage (7%) was for the presence of residue, while the least percentage (1.5%) was for the presence of aspiration. 2% of subjects had incomplete glottic closure and VPV incompetence, 4% of subjects had premature spillage and poor pharyngeal mobility while 4.5% of subjects had penetration and delayed triggering

Age has a significant positive correlation with the total score of dysphagia manifestations with P value of 0.001 and total score of FEES with *p* value of 0.006.

The total score of EAT10 has a significant positive correlation with all the sub-items with *p* values of < 0.001and the total score of dysphagia manifestations with *p* value of 0.001 as shown in Table 7.

**Table 7:** Correlation between total score of EAT10 and sub-items and the total score of dysphagia manifestations

	**Total score of EAT10**	
	**R**	***P* value**
Dysphagia	0.569	< 0.001
Anorexia	0.657	< 0.001
Weight loss	0.484	< 0.001
Salivation	0.394	< 0.001
Accumulation of food in mouth	0.364	< 0.001
Sticking of food in throat	0.783	< 0.001
Nasal regurgitation of fluids	0.284	< 0.001
Cough on food	0.649	< 0.001
Cough on fluids	0.585	< 0.001
Need water to swallow	0.666	< 0.001
Need to multiple swallows	0.587	< 0.001
Need to cut food into small pieces	0.582	< 0.001
Total score of dysphagia manifestations	0.816	0.001

Non-significant *P* value > 0.05, Significant *P* value < 0.05, highly significant *P* value < 0.01

Table 7 shows that the total score of EAT10 has a significant positive correlation with all the sub-items with *p* values of < 0.001and the total score of dysphagia manifestations with *p* value of 0.001

The total score of FEES has a significant positive correlation with salivation with *p* value 0.027, cough on food with p value 0.044 and the need to cut food into small pieces with *p* value 0.013 and total score of dysphagia manifestations with *p* value 0.012 as shown in Table 8.

**Table 8:** Correlation between total score of FEES and sub-items of dysphagia manifestations in the suspected group

	**Total score of FEES**	
	**R**	***P* value**
Dysphagia	0.282	0.329
Anorexia	0.492	0.074
Weight loss	0.492	0.074
Salivation	0.587	0.027
Accumulation of food in mouth	0.442	0.114
Sticking of food in throat	0.073	0.803
Nasal regurgitation of fluids	-0.337	0.239
Cough on food	0.544	0.044
Need water to swallow	0.165	0.573
Need to multiple swallows	0.399	0.157
Need to cut food into small pieces	0.641	0.013
Total score of dysphagia manifestations	0.651	0.012

Non-significant *P* value > 0.05 Significant *P* value < 0.05 highly significant *P* value < 0.01

Table 8 shows that the total score of FEES has a significant positive correlation with salivation with *p* value 0.027, cough on food with *p* value 0.044 and the need to cut food into small pieces with *p* value 0.013 and total score of dysphagia manifestations with *p* value 0.012

The total score of bedside evaluation has a significant negative correlation with consistency of meal with p value 0.015 and positive correlation with presence of difficult consistency with *p* value < 0.001and the most difficult consistency with p value 0.036. Elderly subjects who identified their most difficulty consistencies, and who adapted their environment for meals and ate their easiest consistencies had better bedside tests than those who didn't adapt and ate their difficult consistencies as shown in Table 9.

**Table 9:** Correlation between total score of bedside evaluation and subitems of eating habits

	**Total score of Bedside evaluation**	
	**r**	***P* value**
No. of meals	0.091	0.202
Consistency of meal	-0.172	0.015
Eating environment	0.058	0.415
Presence of difficult consistency	0.363	< 0.001
The most difficult consistency	0.234	0.036

Non-significant *P* value > 0.05 Significant *P* value < 0.05 highly significant *P* value < 0.01

Table 9 shows that total score of bedside evaluation has a significant negative correlation with consistency of meal with *p* value 0.015 and positive correlation with presence of difficult consistency with *p* value < 0.001and the most difficult consistency with *p* value 0.036

Cronbach’s alpha was quite reliable in total score of dysphagia manifestations (0.472) and total score of bedside evaluation (0.451). It was reliable in the total score of eating habits (0.785), total score of EAT10 (0.721) and the total score of FEES (0.696).

**Fig. 1:**
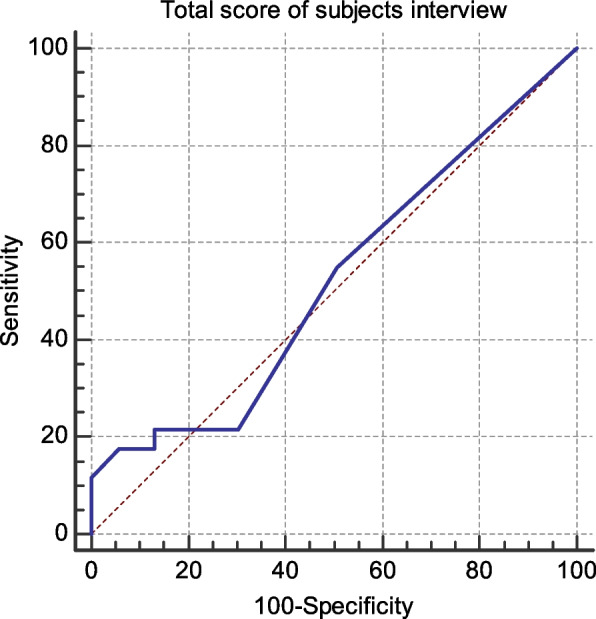
shows cut off point, sensitivity and specificity of total score of symptoms of dysphagia manifestations according to EAT10. The cutoff point of total score of dysphagia manifestations > 5. Its sensitivity is 17.65% Its specificity is 94.20%. The positive predictive value is 69.2%. The negative predictive value is 60.7%

Cut off point, sensitivity and specificity of total score of symptoms of dysphagia manifestations according to EAT10. The cutoff point of total score of dysphagia manifestations > 5. Its sensitivity is 17.65%, specificity is 94.20%, positive predictive value is 69.2% and the negative predictive value is 60.7% as shown in Fig. 1.

**Fig. 2:**
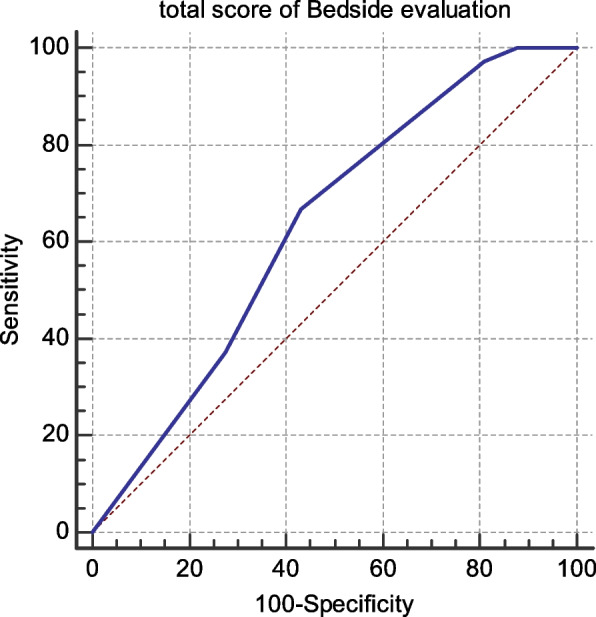
shows cut off point, sensitivity and specificity of total score of bedside evaluation according to EAT10. The cutoff point of total score of Bedside evaluation ≤ 1. Its sensitivity is 66.9% Its specificity is 56.9%. The positive predictive value is 79.2%. The negative predictive value is 41.3%

Cut off point, sensitivity and specificity of total score of bedside evaluation according to EAT10. The cutoff point of total score of Bedside evaluation ≤ 1. Its sensitivity is 66.9%, specificity is 56.9%, The positive predictive value is 79.2% and the negative predictive value is 41.3% as shown in Fig. 2.

## Discussion

Oropharyngeal dysphagia can give rise to clinically relevant complications [29]. When a decrease in swallowing safety occurs, aspiration leads to pneumonia in 50% of cases [30], with a mortality rate of up to 50% [31]. Impaired safety also affects the ability of patients to ingest all the needed calories and water that help them to be adequately nourished and hydrated [30]. The affected elderly people are not always aware of their swallowing dysfunction. OD is considered a geriatric syndrome [32] due to its high prevalence and its relation with various risk factors with consequent poor outcomes. The European Society for Swallowing Disorders and the European Union of Geriatric Medicine Society have recognized it as a geriatric syndrome in a paper published in 2016 [10], confirming its importance and to increase the awareness among physicians and in the society [23]. The difference between physiological swallowing in elderly people and when the aging changes represent an impaired function that is very difficult to judge [33].

The main message of the current study was that OD should be diagnosed early and promptly treated in this population in order to avoid nutritional and respiratory complications.

In Egypt, dysphagia risk screening is not mandatory and the knowledge of prevalence of malnutrition in older adults is lacking. The clinical practice that relies on only single question of what about dysphagia was found in the literature to have low non predicative value although it had other good psychometric values such as sensitivity, specificity and positive predictive value. However, standardized questionnaires help the retrieval of similar information from all patients and prevents essential information omission [17].

Content validity was put in consideration by collecting the common symptoms of dysphagia in this population found in both the literature and the clinical experience of phoniatricians dealing with dysphagia problems in similar age groups. So content validity was considered for comprehensive sampling of the items of the prepared questionnaire. It has been adequately translated and culturally adapted to address the purpose for which it was developed.

This study was performed to assess the presenting symptoms related to early detection of dysphagia in elderly adults even in non-complaining elderly adults and the recognition of the underlying physiological breakdown in their swallowing mechanism.

The screening tool duration ranged between 25 to 30 min which is relatively longer compared to previous screening tools widely used such as questionnaires or bedside evaluation alone. It was important to devise a detailed and comprehensive questionnaire to pick up any subtle dysphagia symptoms in such non complaining population in addition to the use of other objective evaluations such as the bedside and the oral motor assessments to compare and correlate with the responses of the subjects.

Subtle swallowing difficulties faced by the subjects under study were detected as shown in Table 1. More than 36.5% of participants reported cough on fluids, 26.5% of them reported sticking of food in the throat and the need of water to swallow while the least percentages were for the presence of salivation 12.5% and weight loss 15%. These results could be due to the aging related muscle weakness, neuromuscular incoordination, their limited strength and range of movement of several organs involved in the feeding and swallowing process that led to difficulty with feeding and might thereafter have a negative impact on their desire for food intake and subsequently lead to decrease in body weight. Weight loss was previously documented in Nursing home residents in a study by Dell'Aquila et al. [34] in their dysphagic and non dysphagic groups with higher percentage in the dysphagic group. Among the aging-related diseases, two pathological conditions existed in elderly people including sarcopenia and dysphagia. They cause dehydration and malnutrition in this category of population. “Sarcopenic dysphagia” is characterized by swallowing impairment due to the mass and power of swallowing muscles’ loss and might be related to poor oral health status, too. Aging process is strictly related to poor oral health status due to direct impairment of the immune system and wound healing and both physical and cognitive impairment that might indirectly affect elderly people’s ability to do adequate oral hygiene. Therefore, poor oral health could affect nutrient intake, causing malnutrition and frailty [35].

The subjects in the study didn’t either experience difficulty to initiate swallow or nasal regurgitation of food. This was rather expected and could be justified because the subjects under study were not a diseased group and the ones suffering from neurological disorders were excluded prior to the study.

This study showed, that also dysphagia is a major contributor to the reduced food intake in elderly. This is a relevant finding, because dysphagia can be modified with specific measures such as speech and language therapy.

The findings of the current study are in agreement with previous studies as Morley [36] who believed that normal aging shows a decline in food intake known as the anorexia of aging. The fundus of the stomach is less compliant, allowing a greater filling of the stomach antrum that triggers signals to the central nervous system to cease eating. changes to appreciation of taste and smell may reduce hunger drive while tooth loss may impact food texture choices [37]. Thus, a decrease in food intake may predispose to vulnerability for muscle wasting [38].

The data obtained on the eating habits for the sample under study as seen in Table 2 shows that 33% of the participants had 2 meals a day, all of them spend less than 30 min as duration for meal, 34% of them cannot eat all consistencies of food, half of the subjects need prepared environment during eating. 61% of the subjects recognized having difficult food consistency, 97.5% of them had difficulty in eating solids while semisolids were the easiest consistency. Although the number of meals and the meal duration weren’t significant and didn’t indicate a problem but the problems were related to difficulty with certain food consistencies like solids.

The previous results could be explained by the absence of dentition, reduced salivation, decreased range of motion of oral structures and alteration to muscles' strength required for chewing and bolus formation that might be the factors contributing to the difficulty faced with certain types of food especially the solid consistency. This was reviewed at mealtimes, difficulties associated with functional chewing; bolus formation and positioning were noted in frail as opposed to robust elderly [39].

Dysphagia is often associated with difficulties in managing solids as well as liquids. Intake problems associated of eating and drinking has been shown in frail elderly**.** Large solid bolus improperly chewed can fatally occlude the airway [40].

Difficult foods that are clarified in the literature to consistently cause choking and reported on autopsy include meat, bread, and toast. All the previously mentioned types of food have complex structure of fibers that shows difficulty to be broken down effectively by rotary chewing movement using molar teeth. They require Sufficient stamina and also more than 20 chewing strokes per bolus to be efficiently prepared [41].

The previous result may encourage phoniatricians /speech & language pathologists to give instructions and directions to elderly and their caregivers about strategies and mechanisms that can help prevent OD in this vulnerable population, such as taking good care of teeth, maintain good oral hygiene, chewing carefully and taking small bites, and tucking chin down to chest before swallowing to protect the airway. In addition to increasing their awareness about the new advancement of some therapies as ozone therapy that represents a promising treatment option for its ability to modulate inflammation that is caused by limited immunity and exposure to infection related to malnutrition [42], in addition to promoting cartilage growth, and joint repair mechanisms that might be affected by frailty and aging physiological mechanisms [43].

Regarding percentages of items of general and oral motor examination for the sample under study as seen in Table 3, it was found that 12% of subjects under study showed small atrophic tongue and poor muscle power while 3% of them had tongue tremors, and 47% of subjects showed missing teeth.

These oral motor changes that occurred in aging people under study reflected negatively on their swallowing ability as mentioned before such as decrease in the desire for food intake, difficulty in swallowing solids and subsequently decrease in body weight. Missing teeth prevents good preparation of food to be swallowed in the oral cavity with increased risk of residue and post swallow aspiration. This is in agreement with the fact that loss of teeth affects the selection of food of reduced consistency and consequent loss of pleasure in eating clarifying the relation between loss of teeth and poor nutritional status of elderly [44, 45].

This explanation goes with Machida et al. [46] who reported that high levels of sarcopenia are associated with decreased tongue strength and with Butler et al. [47] who mentioned that a reduction in tongue strength has been associated with an increased risk for swallowing difficulties such as aspiration as it increases the likelihood of bolus retention in the pharynx. Impaired oral health can lead to malnutrition and sarcopenia, which can, in turn, cause dysphagia, resulting in a negative cycle that worsens the patient’s general condition [35].

Results mentioned in Table 4 clearly demonstrate that the **s**ubjects under study showed difficulties during bedside evaluation in the form of salivation, accumulation of certain consistencies ‘semisolids, solids and mixed consistency’ represented in 3.5% of them, voice change in 6.5%, difficulty on chewing solids in 27% and chewing difficulty on mixed consistency was found in 52.5% of subjects under study.

It is logical to assume that many of the muscles involved in swallowing may also be affected by aging, even in the absence of other underlying health issues. The muscles of the oropharynx are skeletal, striated muscles and therefore, age-related loss and atrophy might be expected in these muscles, similar to that seen in the limb muscles, despite the fact that the muscles of the head and neck are not weight bearing however, fine control and coordination and a high degree of efficiency is required to undergo the rapid and complex cascade of oro-pharyngeal swallowing.

Solids and mixed consistency food need more muscle power, effort and co-ordination. Mixed consistency foods are popular among the aging people as they depend on them in their meals to save time, effort and ensure balanced intake of nutrients.

Biomechanically a small study of normal adults by Saitoh et al. [48] showed that chewing and initial bolus consistency each changed the relationship between the transport and initiation of swallow, and that when solid and liquid phases food are consumed, a portion of food commonly reach the hypopharynx well before the onset of swallow thus increases the risk of aspiration.

The accumulation of food can be interpreted by Turley and Cohen [49] who stated that oropharyngeal dysphagia in older people is related to impaired swallow efficacy and/or safety due to weak tongue propulsion and prolonged and delayed the oropharyngeal swallow response (OSR). Impaired swallow efficacy is associated with reduced bolus propulsion due to weak muscular tongue force related to sarcopenia [50] leading to accumulation of different consistencies in oral cavity and increase risk of aspiration pneumonia.

The voice changes in aging people can be interpreted by the structural and the physiological changes occur in old age in all systems including those responsible for voice production; the respiratory and vocal tract.

The results in Table 4 showed that voice changes happened in the context of the bedside evaluation, meaning voice changes that occurred directly after oral intake of food and fluids. In this context, the voice changes are a symptom of laryngeal penetration and therefore a major symptom of severe dysphagia.

The changes of calcification and ossification of cartilages, muscles and vocal fold atrophy, vocal fold bowing, reduced mucosal wave and pulmonary lung pressures, volumes, and elasticity have an impact on voice production and quality [51].

For confirmation of presence of dysphagic symptoms and signs in this study, EAT10 a valid self-perceived questionnaire was carried out for all subjects under study and FEES was performed for suspected cases only.

The results obtained for the EAT10 as seen in Table 5 revealed that 12.5 to 17.5% of subjects under study had difficulties related to weight loss, affection of eating pleasure, extra effort with pills and 7.5% of subjects had stress on swallowing. The findings of this self-rating questionnaire confirmed the presence of swallowing difficulties in non-complaining subjects under the study.

Many people recognize such difficulties, assuming they are typical of their aging process that they have to accommodate to. The adaptation occurs gradually that people are unaware they are making compensations.

The results of the FEES as represented in Table 6 revealed that: Subjects showed some difficulties during FEES evaluation as the presence of residue represented in 7%, the presence of aspiration represented in 1.5%, incomplete glottic closure and VPV incompetence represented in 2%, premature spillage and poor pharyngeal mobility in 4% while 4.5% of subjects had penetration and delayed triggering.

This could be explained by age related structural changes that had an effect on swallowing mechanism as in agreement with Aronson [52] who mentioned that the presence of anatomical and physiological changes as an aging process affects both eating and functions of swallowing. Ossification of bones and cartilages like hyoid and thyroid, laryngeal muscles atrophy, laryngeal mucosa dehydration, bowing of vocal folds and loss of laryngeal ligaments elasticity are observed with aging.

All of the previously mentioned changes have a negative effect on both hyolaryngeal excursion and closure of laryngeal vestibule. Even with subjectively adequate chewing function, decreased muscle power and teeth loss limit food choices and chewing efficiency leading to occurrence of post swallow oral residue. Mild delay in the swallow reflex triggering and limited power of the tongue increase pharyngeal residue contributing to frequent trials of swallows clearing [53].

Pelletier [37] stated that the duration of laryngeal excursion and closure is retained until about 60 years of age and then declines. The general slowing of the aging eating and swallowing system mimics age related slowness seen in other systems, such as gait and mobility. Sensory changes are also apparent such as a reduction in olfaction appreciation (smell) and diminished taste.

Another study found a link between frailties with swallowing safety was done by Rofes et al. [32]. Their study showed that more than two thirds of sample under their study presented with oropharyngeal residue, more than half presented with laryngeal penetration of the bolus and 17% demonstrated tracheobronchial aspiration. Impaired tongue propulsion and delayed hyolaryngeal excursion was linked to oropharyngeal residue across liquid thickness levels. These features were not evident in healthy controls.

The previous findings which were found in oral motor examination of the subjects under the current study as muscle weakness especially in tongue muscles can explain the premature spillage and this in agreement with Pitts et al. [13] who mentioned that several physiologic changes associated with aging impact these processes, including loss of muscle mass and function, decreased tissue elasticity, cervical spine changes, decreased saliva production, and reduced compensatory capacity of the brain. Holistically, aging slows deglutition and reduces its efficiency.

It was noted that the muscular changes reduce the effectiveness of expulsive airway behaviors, such as coughing and sneezing, providing a mechanism that increases potential risk for development of pneumonia. Fatty infiltration of skeletal muscle also causes weakness by reducing the physical integrity of the muscle and is also a marker of aging and frailty [54].

Age had positive significant correlation with total score of dysphagia manifestations, and total score of FEES. This finding pointed out to that aging is one of the factors that affect the swallowing efficacy and safety and that the older the age, the more the dysphagia symptoms and findings and the risk of OD increases. With increased life expectancies of the aging populations, this finding increases the importance of increasing awareness of caregivers and health care professionals to OD and its various screening tools.

This can be explained by the several studies which stressed on normal aging that is associated with reduced pharyngolaryngeal sensory discrimination and a higher threshold to trigger the pharyngeal phase as mentioned by Humbert et al. [55]. Elderly adults recruit far more cortical regions during swallowing, suggesting that more cortical involvement is necessary to complete the same swallowing task [56]. Also, Teisman et al. [57] mentioned that healthy elder adults present with prolonged oropharyngeal phase with aging, delay before the onset of the pharyngeal swallow response and increased residues in the pharynx. Wick et al. [58] found that there is a high correlation between absent teeth, ill-fitting dentures, dental disease and sudden choking deaths.

Correlation between total score of EAT10 and the total score of the questionnaire of dysphagia manifestations and scores of its sub items showed that total score of EAT10 has significant positive correlation with the total score and all the subitems of dysphagia manifestations as seen in Table 7.

The results in Table 8 represented significant positive correlation between total score of FEES and sub items of dysphagia manifestations in the suspected group. This adds to the reliability of the items selected in the current protocol as being correlated with a reliable and valid questionnaire as EAT 10 and the instrumental evaluation of FEES.

Baijens et al. [10] explained the importance of FEES who stated that flexible laryngoscopy can reveal important findings, such as pooling in the vallecula, vocal fold immobility, laryngeal or hypopharyngeal masses, or incomplete glottis closure. The authors noted that more than 50% of the patients presenting with dysphagia have a positive finding on laryngoscopy ranging from pooling in the pyriform sinuses to glottic gap to vocal fold paralysis.

It is very important to get correlation between different aspects of subjective evaluation on one hand and the instrumental evidence of swallowing dysfunction or disruption of swallowing process. This is in line with Sakai et al. [59] who stated that many research groups have reported on non-VF swallowing parameters in relation to the swallowing status of healthy older adults. However, as reported in a systematic review made by Madhavan et al. [60], these studies lack the direct comparison or correlation with instrumental evidence of disruption to swallowing function.

The correlation between total score of bedside evaluation and sub items of eating habits as seen in Table 9 showed that total score of bedside evaluation has negative significant correlation with consistency of meal and positive correlation with presence of difficult consistency, and the most difficult consistency. This highlights the importance of good history taking as in the detailed questionnaire of eating habits in the evaluation process of swallowing dysfunction.

For the Reliability Statistics (Cronbach’s alpha) of total score of questionnaires, total score of bedside evaluation and total score of FEES, Cronbach’s alpha was quite reliable in total score of dysphagia manifestations and total score of bedside evaluation. It was reliable in total score of eating habits, total score of EAT10 and total score of FEES.

In the best practice guidelines suggested by many societies, it is recommended that whenever possible, a FEES should be conducted additionally, to provide a comprehensive assessment. This procedure is also recommended in the guidelines for neurogenic dysphagia provided by the German association for neurology [61].

In the current study, the cut off points were carried out against EAT 10. The EAT-10 has been proved to be an excellent tool for the subjective assessment of dysphagia. It is an easy and quick method to give an idea about how patients perceive their swallowing problem. It has shown an excellent internal consistency and test–retest reproducibility [23].

Older people with dysphagia manifestations score > 5 or with a total score of bedside evaluation ≤ 1 should be considered for further swallowing assessment. The subjects in this study had lower mean scores than the cutoff points mentioned in the previous studies. This raises an interesting point for future research on larger scale in the geriatric population and then among different groups of various causes of dysphagia.

Based on the results of sensitivity and specificity in Figs. 1, 2, it was found that the dysphagia manifestations questionnaire can exclude the negative cases more than detect the positive cases. Hence the bedside evaluation is more sensitive than the dysphagia manifestations questionnaire, so it adds to our preference to combine and use both of them in the screening tool to add more accuracy. Dysphagia manifestation questionnaire can predict nearly 70% of the positive results and about 61% of the negative results. The bedside evaluation can predict about 80% of the positive results and about 41% of the negative results.

The current study reported the presence of elderly people who had signs of oropharyngeal dysphagia in nursing homes in the Egyptian community and this is in agreement with previous studies [62–65]. Based on the findings of the current study, an adequate management of elderly people should include oropharyngeal rehabilitation, oral health treatment, and nutritional supplementation to address any age-related functional decline in order to improve the quality of life [35, 66]. Applying screening tool in elderly people would help early identification of those who are at risk of oropharyngeal dysphagia and this is essential for effective management to minimize malnutrition, dehydration, and pneumonia [62].

The comprehensive questionnaire and bedside evaluations in addition to the comparison by a subjective as well as an objective evaluation gives strengths to the results of this study. Also devising Arabic easy to apply dysphagia screening tool that can be used by non-professionals and caregivers widens the field of its applicability in Arabic speaking countries and patients. The limited number of subjects used is a limitation that should be put into consideration for further studies. Another limitation to this study is that FEES was only conducted on suspected cases. Thus, it allowed estimating the difficulties in only subjects with salient symptoms of dysphagia. This way, we tested the specificity of the screening tool and not the sensitivity. However, applying the FEES on the whole sample will reveal those with silent symptoms allowing for proper estimation of apparently normal elderly people with signs of dysphagia.

## Conclusion and recommendation

In order to provide caregivers and practitioners dealing with elderly individuals that are at risk of OD, a comprehensive screening tool, subjective questionnaires as well as objective tests must be included. Dysphagia manifestations questionnaire as well as the Eating habits questionnaire were found to be quite reliable and its items were significantly correlated with the valid and reliable EAT10 questionnaire and some aspects of the instrumental evaluation of FEES as well as the bedside evaluation. 

Eating habits questionnaire was found to be reliable and showed a reliability of 0.785 that is slightly higher than EAT10 questionnaire in this population. The Questionnaire of eating habits is significantly correlated with bedside evaluation and helps to complete the profile of the patients along with dysphagia manifestations questionnaire. Oral motor examination and bedside evaluation helped to confirm the self-perceived symptoms in the subjects under study.

The findings of the current study signify the clinical validity of the prepared questionnaire in assessing swallowing problems for elderly with dysphagia. It helps multidisciplinary teams in early detection of cases with suspected OD, to direct them to specialties for a complete work up for dysphagia to confirm OD and to carry out comprehensive assessment and set an individualized intervention plan preventing OD complications, thus reducing the economic and societal burden and improving patient quality of life.

Older people with dysphagia manifestations scoring > 5 or with a total score of bedside evaluation ≤ 1 should be considered for further swallowing assessment. The subjects in this study had lower mean scores than the cutoff points mentioned in the previous studies. This raises an interesting point for future research to apply the questionnaire on larger sample size of the geriatric population and then among different groups of various causes of dysphagia.

An easy applicable, quick, available and comprehensive tool is essential to use in the geriatrics population even those not complaining of dysphagia to improve quality of life and prevent life threatening complications that can develop later. The current screening protocol might be beneficial to be used by caregivers (especially the questionnaire part) and by the general practitioners.

Further research is needed in this area to expand the knowledge and perception of phoniatricians, thus paving the way for evidence-based improvements to the interdisciplinary team management protocol.

## Data Availability

The datasets used or analyzed during the current study are available from the corresponding author on reasonable request.

## Abbreviations

FEES: Fiberoptic Endoscopic Evaluation of Swallowing
OD: Oropharyngeal dysphagia
ICD: International Classification of Diseases
WHO: World Health Organization
